# Supplementary material for: Uneven terrain treadmill walking in younger and older adults
Source: PLoS One. 2022 Dec 19;17(12):e0278646. doi: 10.1371/journal.pone.0278646 (PMC9762558; doi:10.1371/journal.pone.0278646)
Supplement: S1 Table — (PDF) [file pone.0278646.s002.pdf]

S2 Table. Statistical model results for step duration (s) after accounting for walking speed.

	Value	Std. Error	DF	t-value	p-value	Sig.	ES
**Intercept**							0.14
HFOA, Flat	0.131	0.0554	200	2.36	0.0192	*	
**Group**							0.46
YA	-0.259	0.0871	200	-2.97	0.0033	*	
LFOA	0.369	0.0756	200	4.89	0.0000	*	
**Terrain**							0.11
Low	-0.079	0.0783	200	-1.01	0.3157		
Medium	-0.100	0.0783	200	-1.28	0.2033		
High	-0.142	0.0783	200	-3.37	0.0706		
**Interaction**							0.06
YA Low	0.089	0.1231	200	0.73	0.4688		
YA Medium	0.096	0.1231	200	0.78	0.4371		
YA High	0.111	0.1231	200	0.90	0.3675		
LFOA Low	0.020	0.1074	200	0.18	0.8558		
LFOA Medium	0.037	0.1069	200	0.35	0.7280		
LFOA High	0.069	0.1074	200	0.64	0.5201		

DF, degrees of freedom; ES, Effect Size; HFOA, higher-functioning old adults; LFOA = lower-functioning old adults; YA, young adults.
