# Supplementary material for: Uneven terrain treadmill walking in younger and older adults
Source: PLoS One. 2022 Dec 19;17(12):e0278646. doi: 10.1371/journal.pone.0278646 (PMC9762558; doi:10.1371/journal.pone.0278646)
Supplement: S2 Table — (PDF) [file pone.0278646.s003.pdf]

S3 Table. Statistical model results for step duration variability (%) after accounting for walking speed.

	Value	Std. Error	DF	t-value	p-value	Sig.	ES
**Intercept**							0.11
HFOA, Flat	-1.41	0.747	200	-1.89	0.0600		
**Group**							0.46
YA	-3.36	1.175	200	-2.86	0.0047	*	
LFOA	5.06	1.020	200	4.96	0.0000	*	
**Terrain**							0.22
Low	1.66	1.057	200	1.57	0.1182		
Medium	2.16	1.057	200	2.04	0.0422	*	
High	3.86	1.057	200	3.65	0.0003	*	
**Interaction**							0.15
YA Low	0.17	1.661	200	0.10	0.9204		
YA Medium	0.97	1.661	200	0.59	0.5583		
YA High	0.33	1.661	200	0.20	0.8411		
LFOA Low	0.96	1.448	200	0.66	0.5071		
LFOA Medium	1.79	1.442	200	1.24	0.2151		
LFOA High	3.20	1.448	200	2.21	0.0284	*	

DF, degrees of freedom; ES, Effect Size; HFOA, higher-functioning old adults; LFOA = lower-functioning old adults; YA, young adults.
