# Supplementary material for: Uneven terrain treadmill walking in younger and older adults
Source: PLoS One. 2022 Dec 19;17(12):e0278646. doi: 10.1371/journal.pone.0278646 (PMC9762558; doi:10.1371/journal.pone.0278646)
Supplement: S3 Table — (PDF) [file pone.0278646.s004.pdf]

S4 Table. Statistical model results for anteroposterior excursion variability (%) after accounting for walking speed.

	Value	Std. Error	DF	t-value	p-value	Sig.	ES
**Intercept**							0.02
HFOA, Flat	-0.62	1.84	200	-0.34	0.7327		
**Group**							0.64
YA	-13.45	2.89	200	-4.66	0.0000	*	
LFOA	16.21	2.51	200	6.46	0.0000	*	
**Terrain**							0.21
Low	3.66	2.60	200	1.41	0.1603		
Medium	5.15	2.60	200	2.04	0.0422	*	
High	9.03	2.60	200	3.47	0.0006	*	
**Interaction**							0.04
YA Low	-0.81	4.09	200	-0.20	0.8421		
YA Medium	-0.17	4.09	200	-0.04	0.9664		
YA High	-2.10	4.09	200	-0.52	0.6071		
LFOA Low	0.21	3.56	200	0.06	0.9522		
LFOA Medium	0.02	3.55	200	0.01	0.9949		
LFOA High	-1.60	3.56	200	-0.45	0.6531		

DF, degrees of freedom; ES, Effect Size; HFOA, higher-functioning old adults; LFOA = lower-functioning old adults; YA, young adults.
