# Supplementary material for: Uneven terrain treadmill walking in younger and older adults
Source: PLoS One. 2022 Dec 19;17(12):e0278646. doi: 10.1371/journal.pone.0278646 (PMC9762558; doi:10.1371/journal.pone.0278646)
Supplement: S4 Table — (PDF) [file pone.0278646.s005.pdf]

S5 Table. Statistical model results for mediolateral excursion variability (%) after accounting for walking speed.

	Value	Std. Error	DF	t-value	p-value	Sig.	ES
**Intercept**							0.14
HFOA, Flat	-2.84	1.22	200	-2.33	0.0210	*	
**Group**							0.26
YA	-1.98	1.92	200	-1.03	0.3038		
LFOA	5.57	1.67	200	3.34	0.0010	*	
**Terrain**							0.27
Low	2.76	1.73	200	1.59	0.1127		
Medium	5.55	1.73	200	3.21	0.0016	*	
High	7.23	1.73	200	4.18	0.0000	*	
**Interaction**							0.08
YA Low	-0.02	2.72	200	0.01	0.9928		
YA Medium	-2.36	2.72	200	-0.87	0.3855		
YA High	-2.45	2.72	200	-0.90	0.3682		
LFOA Low	1.02	2.37	200	0.43	0.6670		
LFOA Medium	-0.95	2.36	200	-0.40	0.6881		
LFOA High	-0.58	2.37	200	-0.24	0.8070		

DF, degrees of freedom; ES, Effect Size; HFOA, higher-functioning old adults; LFOA = lower-functioning old adults; YA, young adults.
