# Supplementary material for: Uneven terrain treadmill walking in younger and older adults
Source: PLoS One. 2022 Dec 19;17(12):e0278646. doi: 10.1371/journal.pone.0278646 (PMC9762558; doi:10.1371/journal.pone.0278646)
Supplement: S5 Table — (PDF) [file pone.0278646.s006.pdf]

S6 Table. Statistical model results for perceived stability ratings.

	OR	2.5%	97.5%	Sig.
**Group**				
YA	1.22	0.721	2.05	
LFOA	2.83	1.798	4.50	*
**Terrain**				
Low	4.15	1.799	10.82	*
Medium	16.96	7.830	42.56	*
High	68.03	31.010	172.67	*

LFOA = lower-functioning old adults; OR, odds ratio (relative to higher-functioning older adult group or Flat terrain); YA, young adults.
If odds ratio > 1, the effect is in the direction of less stable walking.
If odds ratio < 1, the effect is in the direction of more stable walking.
If odds ratio confidence interval [2.5%, 97.5%] contains 1, the effect is not significant.
