# Supplementary material for: Uneven terrain treadmill walking in younger and older adults
Source: PLoS One. 2022 Dec 19;17(12):e0278646. doi: 10.1371/journal.pone.0278646 (PMC9762558; doi:10.1371/journal.pone.0278646)
Supplement: Attachment — Submitted filename: REVIEWER_PONE-D-22-08783_ReviewerComments_020822.pdf [file pone.0278646.s008.pdf]

PLOS ONE

Submission ID: PONE-D-22-08783

Reviewer’s comments

In the submitted study, the author(s) tested young adults, high-, and lower-functioning elders in terms of gait kinematic variability when walking on four different terrains. Results revealed that the changes in terrain unevenness on a treadmill increased gait kinematic variability and reduced perceived gait stability.

There are a number of topics that need to be addressed, as presented in the following General and Specific Comments.

General Comments

- There is a mix of broader information and a lack of elaboration of research evidence concerning the mechanisms involved and the examined variables in the Introduction. It is recommended to re-organize the contents of the Introduction and to highlight the specific aim of the submitted study.
- An EEG setup is mentioned in Materials and Methods (L74-77), but no further details and data are presented. Is this correct? If yes, what was the rationale to include EEG recordings?
- Effect sizes should be reported in the respective descriptive results Tables.
- Provide additional research evidence to support the arguments about the mechanisms involved in the altering gait due to the experimental conditions in the Discussion.

Specific Comments

Abstract

- L20-25: There is a constant use of ‘We’ at the initiation of the sentences. It is recommended to rephrase.

Introduction

- See the respective General Comment.
- L39-44: It is proposed to refer to the effects of aging on gait variability since elderly and not disabled participants were tested.
- L45-49: This paragraph refers to a general content and disrupts the logic flow of the Introduction. A presentation of the examined variables could add to the context at this point.
- L53-54: Elaborate on the findings of the cited studies.
- L64-68: Elaborate on the mechanisms that alter gait because of age and uneven ground in order to build a stronger rationale for the study.
- L68-69: See the specific comment for L45-49.

Materials and Methods

- L74-77: See the respective General Comment.
- L80: Based on the demographic data of the young participants, the majority of the younger adults were in their early 20s. Was the inclusion of participants aged 40 yrs old necessary?
- L90-100: It is proposed to delete “so that others may replicate the spatial configuration we used”.

- L110-113: Was the effect of the coloring of the disks on their perception by the participants checked? In specific, as red is mainly related to danger, could it be that the participants were more cautious when negotiating the disks just by gazing the color and not by estimating the disks' size? In addition, was the visual acuity of the participants checked? Finally, was the contrast of the colors of the disks with the color of the belt within a reasonable range?
- L125: Is there further evidence for the selection of the 75% speed?
- L170-190: Provide additional information about the familiarization process, as well as the reliability scores of the used methods.
- L173: Is there an estimate of data extraction accuracy due to the 20 N threshold?
- L176: Define the procedure to identify the outliers.
- L229-248: This part of the manuscript seems more appropriate for the data processing subsection.

<u>Results</u>

- State the effect sizes of the comparisons.
- Report all p-values in a similar manner, i.e., using three decimal digits.
- Not all curves are visible in Figure 4.

<u>Discussion</u>

- See the respective General Comment.
- L445: Cite the mentioned literature.
- L457-460: This should be mentioned as a limitation of the study.
- L491-492: Elaborate on this statement.
- L520: How often did this occur during the measurements?
- L532-533: Please clarify what is meant here.
