# Supplementary material for: Uneven terrain treadmill walking in younger and older adults
Source: PLoS One. 2022 Dec 19;17(12):e0278646. doi: 10.1371/journal.pone.0278646 (PMC9762558; doi:10.1371/journal.pone.0278646)
Supplement: Attachment — Submitted filename: Author Response_2022_10_21.docx [file pone.0278646.s009.docx]

# Editor

**Comments:**

We invite you to submit a revised version of the manuscript that addresses the points raised during the review process.

The reviews are positive but raise a number of questions that should be addressed in a revision. In terms of the study design and analyses, you mention EEG but no data are discussed or presented, it would be good to include effects sizes and to see if more informative ways of plotting the data would be useful. There are also questions about the possible influence of treadmill speed differences and whether using average values for the statistical analysis is an appropriate choice. More details on the use and processing of the fused sensors is requested as well as more evidence to support the arguments about mechanisms that alter gait. Please note also that PLOSONE requires the underlying data to be made available upon publication.

**Response:**

We found the Reviewers’ comments to be very constructive. We have made numerous edits to improve the manuscript, including but not limited to the following. We have added clarity regarding why EEG was mentioned. We now account for the influence of treadmill speed in the statistical analysis and include effect sizes. We provide alternative plotting of the data in new supplemental figures. We now include example code and data regarding our processing of IMU data and we compare it to a motion capture system. Finally, we now better discuss the mechanisms of altered gait with aging and why we chose to focus on kinematic variability measures to quantify gait stability.

# Reviewer 1

**General Comments**

1. There is a mix of broader information and a lack of elaboration of research evidence concerning the mechanisms involved and the examined variables in the Introduction. It is recommended to re-organize the contents of the Introduction and to highlight the specific aim of the submitted study.
   a. Thank you. We revised the Introduction to focus it on the specific aim of the study.
2. An EEG setup is mentioned in Materials and Methods (L74-77), but no further details and data are presented. Is this correct? If yes, what was the rationale to include EEG recordings?
   a. The data were taken from a larger study called *Mind in Motion* which is still ongoing. The larger study aims to answer questions related to the neural control of movement using EEG, fMRI, fNIRS, and clinical assessments. Participants wore EEG to help answer questions from the larger Mind in Motion study, but it is not directly relevant to the present manuscript. Based on the Reviewer’s comment, we have modified the manuscript to better explain why participants wore EEG.
3. Effect sizes should be reported in the respective descriptive results Tables.
   a. Effects sizes are now reported.
4. Provide additional research evidence to support the arguments about the mechanisms involved in the altering gait due to the experimental conditions in the Discussion.
   a. We now discuss that increased kinematic variability as a function of the experimental conditions could indicate the uneven terrain is leading to a loss of stability.

**Specific Comments**

Abstract

5. L20-25: There is a constant use of ‘We’ at the initiation of the sentences. It is recommended to rephrase.
   a. Based on the reviewer’s comment, we have revised the lines to reduce the use of ‘we’ at the beginning of sentences.

Introduction

6. See the respective General Comment.
   a. Please see our response to General Comment #1.
7. L39-44: It is proposed to refer to the effects of aging on gait variability since elderly and not disabled participants were tested.
   a. We have updated the Introduction to refer to the effects of aging on gait.
8. L45-49: This paragraph refers to a general content and disrupts the logic flow of the Introduction. A presentation of the examined variables could add to the context at this point.
   a. We have revamped the Introduction for better logical flow.
9. L53-54: Elaborate on the findings of the cited studies.
   a. We have removed this section in the revised Introduction.
10. L64-68: Elaborate on the mechanisms that alter gait because of age and uneven ground in order to build a stronger rationale for the study.
    a. The revised Introduction now better discusses how aging and uneven terrain alter gait.
11. L68-69: See the specific comment for L45-49.
    a. In the revised Introduction, we better introduce kinematic variability and its significance beforehand (i.e., prior to listing it as our primary outcome measure).

Materials and Methods

12. L74-77: See the respective General Comment.
    a. Please see our response to General Comment #2.
13. L80: Based on the demographic data of the young participants, the majority of the younger adults were in their early 20s. Was the inclusion of participants aged 40 yrs old necessary?
    a. Previous literature suggests that individuals aged in their 20’s and 30’s have no significant difference in gait from each other, so we saw no reason to exclude 30 year olds.
14. L90-100: It is proposed to delete “so that others may replicate the spatial configuration we used”.
    a. Based on the Reviewer’s comment, we have revised the sentence. We also moved the sentence slightly earlier in the paragraph for logical flow.
15. L110-113: Was the effect of the coloring of the disks on their perception by the participants checked? In specific, as red is mainly related to danger, could it be that the participants were more cautious when negotiating the disks just by gazing the color and not by estimating the disks’ size? In addition, was the visual acuity of the participants checked? Finally, was the contrast of the colors of the disks with the color of the belt within a reasonable range?
    a. We colored the disks differently for each condition as part of a larger study. Our intent was to help participants recall differences in the terrain in a separately collected imagined walking task (functional MRI). It is possible that the coloring of the disks influenced walking behavior, but we think the terrain itself is difficult to navigate and is a bigger factor than the coloring. Future studies could re-examine the effect of uneven terrain with uniform coloring across conditions. We checked the visual acuity of the participants with a standard Snellen eye chart as part of the larger study. Participants with severe visual impairment or corrected visual acuity less than 20/40 were excluded. The treadmill belt was black and the colors of the terrain (green, yellow, orange, red) were relatively vibrant so visual contrast should not have been an issue (Fig 1).
16. L125: Is there further evidence for the selection of the 75% speed?
    a. For flat treadmill walking, participants’ self-selected treadmill speed is roughly 15% slower than their overground self-selected speed. We set our initial target treadmill speed to 75% of each participant’s slowest self-selected overground walking speed (25% reduction) to compensate for this anticipated effect. We have updated the Methods and cited literature comparing overground and treadmill walking speeds.
17. L170-190: Provide additional information about the familiarization process, as well as the reliability scores of the used methods.
    a. The familiarization process was previously described in L128-133. Based on the Reviewer’s comment, we have updated the Methods to reference the reliability of the specific hardware we used (Loadsol force sensors and APDM Opal IMU system).
18. L173: Is there an estimate of data extraction accuracy due to the 20 N threshold?
    a. A threshold of 20 N is commonly used in gait event detection. We have updated the Methods to cite the use of 20 N in previous literature.
19. L176: Define the procedure to identify the outliers.
    a. We revised this section of the manuscript for clarity
20. L229-248: This part of the manuscript seems more appropriate for the data processing subsection.
    a. We respectfully disagree.

Results

21. State the effect sizes of the comparisons.
    a. We now report effect sizes
22. Report all p-values in a similar manner, i.e., using three decimal digits.
    a. We have updated all p-values to three decimals.
23. Not all curves are visible in Figure 4.
    a. We were not able to find an issue with the figure ourselves. Are you able to upload an image with your review to highlight the issue?

Discussion

24. See the respective General Comment.
    a. Please see our response to General Comment #4.
25. L445: Cite the mentioned literature.
    a. We added citations for clarity.
26. L457-460: This should be mentioned as a limitation of the study.
    a. We now account for the effect of walking speed, so it is no longer a limitation.
27. L491-492: Elaborate on this statement.
    a. We have revised the paragraph for clarity.
28. L520: How often did this occur during the measurements?
    a. We looked back through our notes and we counted a total of 39 times that an individual piece of terrain came loose from the treadmill. That is 39 total times spread across all walking trials for all 71 participants (i.e., its occurrence was very infrequent). We have updated the manuscript with this information.
29. L532-533: Please clarify what is meant here.
    a. We revised this sentence for clarity.

# Reviewer 2

**General Comments**

1. The authors have presented an interesting and thoughtful experiment investigating how unevenness in terrain influences gait dynamics in multiple groups of participants: young adults and higher and lower functioning older adults. The design of the uneven terrain treadmill is creative and effective, and the experimental protocol and measurements are well thought out and clearly explained. The main outcome measures are clear and straightforward. Increased terrain roughness was associated with a decrease in step duration at a fixed speed.
   a. Thank you.
2. This experiment was clearly designed for within-subjects comparisons. The treadmill speed was based upon self-selected speeds overground, which means that speed varied between subjects and there is probably a difference in average speed among the groups. Considering this, the most relevant statistical comparisons are within-subject changes across terrain conditions. Yet group effects were also analyzed and reported in the text. The authors report that lower functioning older adults tended to use longer step durations. It seems likely that this is mainly a speed effect because the treadmill speed was slower on average for this participant group. It seems completely feasible to simply include speed as an additional continuous co-variate in the statistical model, possibly with a group*speed interaction term. Such an analysis could be useful in enabling quantification of both within-subject and between-group effects.
   a. We now account for the effect of walking speed in the statistics to better quantify differences between groups.
3. An additional statistical point is that the justification for averaging the gait metrics before running statistics is unclear. Linear mixed effects models can handle repeated measures. Two stability ratings were maintained for statistical analysis, so it is not clear why the other data were averaged.
   a. In the case of the continuous measures, it is technically better to average across two trials since it reduces the standard error by a factor of 1/sqrt(n). However, for the stability ratings where only 5 possible categorical values are possible (steady, unsteady, mild/mod/very unbalanced), it is better not to average. This is because it is difficult to define the mean of two categorical variables, even if they are ordinal. For example, if one trial is rated “steady” and the other “unsteady”, it is not clear how to define the average perceived stability of the two trials. Therefore, we averaged the values across trials for the continuous variables but not for the perceived stability rating (ordinal categorical variable).
4. The authors have selected coefficient of variation as the stability metric in this study. While I agree that this is a practical and useful proxy for stability. I think it would strengthen the paper to include a discussion of the reasoning behind this, to make the rationale and validity of this choice clear to readers. (Instead of simply referencing a paper without explanation. )
   a. We have updated the manuscript to better explain why we chose to use gait kinematic variability (coefficient of variation) to quantify stability.
5. Data transparency: In the figures, raincloud plots or dot plots overlaid onto box-and-whiskers would be preferable for data transparency. Violin plots add unnecessary 'ink' to graphs by duplicating my mirroring the distribution, which doesn't provide additional information. Using rainclouds (half a violin plot plus the dot scatter to the side), provides more information with similar inkscape.
   a. We now include raincloud plots. Unfortunately, we could not plot half violin’s (could only plot full violin), but the dot scatter has been added to one side.

**Specific Comments**

6. What was the motivation for the different colors of the uneven terrain disks?
   a. We wanted it to be easier for people to remember walking over the terrain during a separate fMRI imagined walking task (see larger study). Another Reviewer also asked for clarity on the coloring of the disks, so we have added further detail explaining our reasoning in the Discussion.
7. Lines 176-177: How did you determine the outliers in the data- did you have a consistent threshold based on number of standard deviations from the mean?
   a. We identified outliers as individual data points that were 2.5 standard deviations from the mean. Based on the Reviewer’s comments, we revised this section of the manuscript for clarity.
8. Line 180-181: Why average across two trials rather than include two repeats for each subject within the statistical analysis?
   a. Please see response to General Comment #3.
9. Line 184: What method was used for offline synchronization of the IMU with the ground force sensors?
   a. The IMU and ground force sensors were synchronized to each other through the EEG system. Note that EEG analysis is outside the scope of the present study but it is a focus of the larger *Mind in Motion* study. Thus, the EEG hardware served as the primary system, and all data streams were synchronized to EEG. To synchronize the IMU and EEG signals to each other, the IMU system sent a brief 5V square pulse at the beginning and end of each walking trial. The EEG system detected the rising edge of the trigger signal coming from the IMU system (5V pulse) and marked the event timing (start and stop of trial). Given these 2 synchronization events, the time systems of the two recording systems (EEG + IMU) can be related to each other, and the data signals from one system can be expressed in the timebase of another through linear interpolation. The ground force sensors were synchronized to the EEG system in a similar but slightly more complicated manner. Since the ground force sensors (Loadsol) do not allow us to send or receive trigger signals by default, we had to create a custom solution to record an external synchronization signal. Instead of just one left foot sensor and one right foot sensor, we also utilized an extra (third) Loadsol sensor and dedicated it to synchronization. We found that we could reliably cause a square-pulse-shaped pseudo-force signal to appear on the sensor by briefly applying a resistor across two pins on the Loadsol device then removing the resistor. We created a custom circuit based on a solid state relay which uses one control signal (5V pulse) to simultaneously open/close two switches (< 1 ms delay). One switch connects to the EEG system and the other connects to the Loadsol system. In this way we can send brief 5V pulses to the EEG system while simultaneously applying a resistor to the extra Loadsol sensor to create a pseudo-force signal (square pulse). We identify the rising edge of the pseudo-force signal to mark synchronization events in the Loadsol system and pair them with the event markers in the EEG system. Finally we use linear interpolation to relate the two systems and synchronize the data. As part of addressing another Reviewer comment about the validity of the IMU processing, we now include example code showing how to synchronize two data streams.
10. Fig 3: In the IMU processing pipeline, it is not clear why it would be necessary to filter both before and after integration? It seems that the correct bandpass filter before both integration steps should be sufficient. Is the 2nd filtering step getting rid of integration drift (in that case it would only need to be a low-pass filter).
    a. Our primary reason for filtering after integration was to remove integration drift. We now include example data and scripts with ground-truth motion capture marker data so others can test out the effect of filtering the IMU data.
11. Line 231: There is no theoretical limit of 100% for the coefficient of variation. The standard deviation can be larger than the mean. Consider for example a parameter that has a mean value of zero but with a distribution that spans positive and negative values. In that case, the CV would be infinity, which is one reason that this measure can be problematic for variables that have a mean value close to zero.
    a. Thank you for catching this error. In our case, the denominator (mean) is guaranteed to be strictly positive which is why we previously thought the CV should have a bound. However, as it turns out, the numerator (SD) can still become very large relative to the denominator (mean), even when the mean does not equal zero, so the CV is still unbounded from above. Thank you again for catching this. We have updated the manuscript and removed the inaccurate statement.
12. Line 437: Differences between the two studies here could also relate to differences in data filtering techniques.
    a. That is true. We have updated the manuscript to acknowledge that differences between studies could also be due to filtering techniques.
13. Line 405: The comparison to Santuz and colleagues is confusing and seems incorrect as currently stated. If Santuz and colleagues found no change in cadence, then this does not seem similar to the current results. An increase in step duration with increased unevenness (as you found here) would be associated with a decrease in cadence, not a constant cadence, as found by Santuz and colleagues. If you are specifically suggesting that the most comparable conditions between the two studies is the ‘low’ unevenness condition, then this needs to be more clearly stated.
    a. Based on the Reviewer’s comment, we have revised the sentence for clarity.

# Reviewer 3

**General Comments**

1. This study is about demonstrating the feasibility of a novel customizable uneven terrain treadmill by showing how some gait parameters change in three different participant groups relative to the change of the terrain unevenness. I really found the design of the treadmill promising and I also appreciate that the authors used inertial measurement units (IMU) and shoe insoles to quantify the findings rather conventional measures such as force plates and motion capture. I believe the ease of construction of the uneven treadmill and portable measures will yield different studies in gait research and rehabilitation in the future. It’s also promising to see the results of an uneven terrain on different populations so other researchers approach uneven terrain more seriously as a research, rehabilitation,and testing tool.
   a. Thank you for your kind remarks.

**Specific Comments**

2. The authors did not give much detail about how they processed the IMU data to find the sacral position. Why did they use 0.2-20Hz filtering, what happens if these frequencies change? There are two integrations (Fig. 3) is there any drift due to these integrations? Did they integrate for each stride? Is there any correction applied based on constant speed walking or any gait parameter assumptions such as periodicity? How did they define the rough estimate of forward walking direction? When they calculate average orientation why did they use entire trial rather than each stride? How did they define the north (magnetic north from the magnetometers of the IMU?) to calculate the angle between northand forward? How did they synchronize the shoe insoles with the IMU’s? If these details were given before in a separate paper of theirs or some else’s, it would be good to refer to those papers while answering the questions.
   a. The 0.2 Hz high-pass portion of the filter is to remove integration drift. We tried to select a value that was low enough in frequency to not remove the main period of walking (1 / stride duration). The slowest average step duration (across all participants) was approximately 2.5 seconds (0.4 Hz). We did not integrate from stride-to-stride. The rough estimate of the forward direction came from knowing how the axes of the IMU are roughly oriented on the person. The z-axis roughly points backward (Fig 4A), so negative z is roughly forward. We used the entire trial when calculating the average orientation because we wanted to express movement in terms of the treadmill’s reference frame. At any given moment, the person could be facing one of many directions on the treadmill (instantaneous orientation). However, over the long-term, the average orientation of the person should be well-aligned with the treadmill (person faces forward on average). Magnetic north was defined by the magnetometers in the IMU. The IMU software performed sensor fusion (accelerometer, gyroscope, magnetometer) during data collection and logged a quaternion time-series that represents the orientation of the IMU with respect to the world as a function of time (NWU reference frame). Another Reviewer asked about the synchronization process. Please see our response to their comment for detailed information (Reviewer 2, comment 9).
3. If the authors agree to share a sample raw IMU data (might be one trial or a part of it) and a sample script (code) that finds the sacral position, that would be nice and make the manuscript get more citations and would help to answer the questions above. Again, this is only if the authors agree to share.
   a. We now provide example data and matlab scripts so others can replicate our IMU processing approach and adjust the filtering parameters.
4. Do the authors have any validity for the IMU estimates such as comparison of sacral position found from the IMU’s and motion capture? If this is done in another study, they can refer to that experiment and give its stats. Or if possible and would be much better, for 1 or 2 subjects can the authors put a motion capture marker on the sacrum and the IMU, collect some walking data on the flat treadmill(doesn’t have to be long just a couple of short trials of different speeds) and show the correlation anderrors between the sacral position found from the IMU’s and motion capture? If this comparison is given, the readers trust the processing more and they would be more likely to apply this processing in their research.
   a. We now provide example data and matlab scripts to compare IMU estimates with ground-truth motion capture data.
5. The results overall make sense and nice, though not surprising. The authors mostly compared their results with other studies to show the validity of their novel treadmill and processing but did not comment much about what the results mean. I believe more discussion about the meaning of the results would be better. Basically, why is more variability bad or why does it mean less stability?
   a. The review by Bruijn et al. nicely summarizes how gait variability is a valid measure of stability and increased gait variability is related to increased fall risk (over flat terrain). In the Introduction, we now better introduce gait variability. In the Discussion, we also now comment on the meaning of the results by noting that increased kinematic variability (our main result) can indicate there has been a loss of stability and an increased chance of fall.
6. Energetics is a dominant factor in overground human walking, it’s also dominant on uneven terrain[1,2]. On uneven terrain balance in addition to energy consumption balance might also be a dominant factor. If the sensors (vision, vestibular system, proprioceptors, cutaneous receptors so on) and the actuators (muscles) of the body have some depreciation in older adults then very likely their central nervous system is doing its best to do the task (staying on the uneven treadmill) given its depreciated sensors and actuators and this might end up being more variable. If the older adults were somehow forced to be less variable on uneven terrain the fall risk might have been bigger (just an idea). Then Maybe being variable on uneven terrain is good. Is there a study that shows being variable is bad or increase fall risk on uneven terrain? I would like to see author’s comments in the discussion (maybe a short paragraph) about variability and stability in this framework. I know what I’m asking is beyond this paper and further experiments might be required (specific tasks and constraints) to answer (if possible) this question, but some comments in the discussion would be nice and would add more value to the paper.
   a. We now discuss the effect of energetics on uneven terrain walking in the Introduction. We also discuss the effect of weakened muscles and reduced sensory capabilities. Note that we recorded perceived stability along with kinematic variability. Both of these measures point to changes in stability (worsened stability).
7. One other thing is what would happen if the authors were to use some of the stability metrics in the reference 28? Would the terrain height and age make all or some of those metrices worse and briefly why? This is just commenting in the discussion, no analysis.
   a. Using other stability metrics would be interesting for future studies but our focus was to see if a given stability metric changed with uneven terrain.

[1] https://arxiv.org/abs/2207.11224

[2] O. Darici, A. D. Kuo, Humans optimally anticipate and compensate for an uneven step during walking. eLife 11, e65402 (2022)
